# Investigating the Effects of Discrimination Experiences on Everyday Metamemory Accuracy

**DOI:** 10.1093/geroni/igaf122.1308

**Published:** 2025-12-31

**Authors:** Timothy Ly

**Affiliations:** The University of Alabama, Tuscaloosa, Alabama, United States

## Abstract

Lifetime and daily experiences of discrimination contribute to impaired performance on cognitive assessments. However, the underlying mechanism by which discrimination negatively impacts cognition is unclear. Recent research investigating stress-induced impairment of metamemory may address the relationship between discrimination experiences and cognitive impairment. The aim of this study was to determine the relationship of lifetime and daily experiences of discrimination, daily affect balance, baseline objective cognitive performance, and sociodemographic variables (age, race, ethnicity, and sex) with metamemory accuracy across the lifespan (ages 20-75). Impaired metamemory accuracy was defined by the number of subjective cognitive complaints. Diary data from the Midlife in the United States (MIDUS Refresher 1) Daily Diary Project (N = 782) was used for these analyses. Results from linear mixed model analyses showed significant within-person effects of daily discrimination, where people who reported more daily discrimination also reported lower metamemory accuracy, and daily affect balance, where people who reported very negative affect also reported lower metamemory accuracy. Additionally, linear mixed model analyses revealed significant between-person effects of race on metamemory accuracy, with individuals from minoritized racial groups generally reporting poorer metamemory accuracy. Daily discrimination experiences also interacted with other variables in predicting day-to-day metamemory accuracy. These findings add to our understanding of how psychosocial stress in the form of daily discrimination experiences may impair metamemory processes contributing to increased subjective cognitive complaints. Future research should consider the contribution of daily experiences of discrimination across the lifespan to poor cognitive outcomes in later life.

